# Objectively Assessing the Effect of a Messenger-based Intervention to Reduce Sedentary Behavior in University Students: A Pilot Study

**DOI:** 10.1007/s10935-023-00735-1

**Published:** 2023-05-12

**Authors:** Mona Kellner, C. Dold, M. Lohkamp

**Affiliations:** 1 Department of Sports and Sports Science, Heidelberg University, Heidelberg, Germany; 2 Heidelberg University of Education, Heidelberg, Germany; 3 SRH University Heidelberg, Heidelberg, Germany

**Keywords:** University health promotion, Sedentary behavior, M-health, Digital intervention

## Abstract

**Aim:**

Sedentary behavior poses a serious health risk. Students in particular are highly affected by prolonged, uninterrupted periods of sitting due to routines in everyday university life, such as attending lectures, self-study periods in the library, etc. Whereas university students are mostly young and therefore appear to be healthy, evidence-based consequences of prolonged sitting may come to pass in prospective times. Therefore, primary prevention must be initiated to shield university students from the occurrence of non-communicable diseases (NCDs). Consequently, the study aims to evaluate a messenger-based intervention designed to reduce sedentary time among university students.

**Subjects and Methods:**

The effectiveness of the intervention was assessed in a randomized controlled trial with a convenience sample of thirty-four German university students. ActivPal devices (Pal Technologies Ltd., Glasgow) were applied to measure sedentary behavior objectively before and after a 3-week intervention of messages to interrupt sedentary time. An additional evaluation of the messages was carried out.

**Results:**

Sedentary behavior decreased by about one hour in the intervention group. Explorative analysis shows a statistically significant, negative correlation between sedentary time at baseline and the change of sedentary behavior over time in the intervention group (r = − .81) indicating effectiveness of the intervention for the participants with the highest sedentary times at baseline. Additionally, the messages were considered appropriate by the participants.

**Conclusion:**

A reduction of sedentary time of one hour per day in the intervention group is practically significant. The current investigation had similar findings with prior studies where promising results for the reduction of sedentary behavior were observed through mobile-based interventions. The detected effects of the intervention in this pilot study demonstrate an opportunity for further research in this field.

## Introduction

A major work field in public health today is the research on the effects of sedentary behavior on the populations’ health. Sedentary behavior is defined as activities that are performed in a seated or reclined position and do not exceed a maximum energy expenditure of 1.5 metabolic equivalents (MET) (Tremblay et al., 2017). Sedentary behavior triggers various pathophysiological processes, including a reduction in muscular lipoprotein lipase activity, lipid and carbohydrate metabolism, a reduction of insulin sensitivity as well as a decrease in cardiac output and vascular function (Park et al., 2020). Consistent with these explanatory approaches, studies indicate that sedentary behavior increases the risk for cardiovascular diseases, type 2 - diabetes, cancer and mortality risk, showing a dose-response association (Biswas et al., 2015; Ekelund et al., 2016, 2019; Patterson et al., 2018; Zhao et al., 2020). Furthermore, studies suggest a link between long periods of sitting and an increased risk of depression (Kandola et al., 2020; Lee & Kim, 2019). Around the globe, 3.8% of all-cause mortality can be attributed to excessive sedentary behavior (Rezende et al., 2020). Research also outlines, that sedentary behavior is an independent risk factor for detrimental health outcomes (Patterson et al., 2018). Consequently, detrimental effects of sedentary behavior can hardly be mitigated through an increase of physical activity but only through a reduction of the sedentary behavior itself and an isotemporal substitution with moderate to vigorous physical activity (MVPA) (Clarke & Janssen, 2021; Koster et al., 2012).

However, current research has not reached a consensus on a cut-off value for hazardous sedentary behavior yet. The amount of daily sitting time negatively affecting the health of the population varies from 3 h/day (h/d) (Rezende et al., 2020) to 8 h/d. (Schmid et al., 2015) whereas Chau et al. (2013) report a 5% higher risk of all-cause mortality for each additional hour above a daily sitting time of 7 h. Similarly diverging are surveys which assess the prevalence of the sedentary behavior of the adult population worldwide. A global review from 2020 reports a median of 4.7 h daily sedentary time (Mclaughlin et al., 2020). Consistent with these findings are the results of a recent scoping review, reporting a median sitting time of 5 h/d (Rezende et al., 2020). In stark contrast to that, local examinations in the US and in Germany show far higher sedentary times: A US National Cohort Study reports an average daily sitting time in adults of 752.4 min (12.54 h/d) (Duran et al., 2021) and a recent health report of a German health insurance company notes on average 523 min (8.71 h/d) of daily sedentary behavior (Froböse & Wallmann-Sperlich, 2021). Regarding a possible cut-off value starting at 3 h/d all findings reveal worrying sedentary times in the adult population. According to the German health reports’ data, young adults seem to be particularly affected. Compared to other age groups, the population between 18 and 29 years of age show the highest sitting times: the average daily sitting time in this group exceeds 10 h/d and is partly linked to the use of electronic media (2 h/d) (Froböse & Wallmann-Sperlich, 2021). In their systematic review and meta-analysis, Castro et al. (2020) emphasize the particular affliction of university students from long periods of sitting compared with the general population of the same age.

The emphasis of the current contribution lies on the sedentary lifestyle of university students. As far as we know, the everyday life of university students is marked with several activities which require sitting, such as attending university lectures, computer use, doing homework, learning for exams, or writing term papers (Castro et al., 2020). Current research shows that young adults also spend a lot of their leisure time watching TV, using the notebook or their smartphone, to which is commonly referred to as ‘screen time’. Screen time is one of the most prominent activities involving young adults in sedentary behavior (Froböse & Wallmann-Sperlich, 2021; LeBlanc et al., 2017). Since the outbreak of the covid-pandemic university students spend far more time at home on their computers. Remote working became a permanent part of the students’ life, leading to more screen time and less low-intensity physical activity due to the measures to curb the pandemic (Bates et al., 2020; Zieff et al., 2021). Thus, not only the young adults’ leisure time is characterized by a lot of sedentary screen time, but also their everyday student activities.

To counteract the excessive sedentary behavior which evolved from remote working in pandemic times a digital, messenger-based intervention was developed to reduce sitting time in university students.

As the intervention particularly aims to reduce sedentary behavior in university students, who seem to frequently use their smartphone and messenger services, a messenger-based intervention seems to be an effective way to reach the target group. Scientific evidence of the effectiveness of text-messages can be found in various fields of public health promotion, such as smoking cessation (Head et al., 2013). Additionally, a recent review revealed that messenger-based interventions can increase physical activity in children and adolescents (He et al., 2021). However, only little, but promising evidence is available for the reduction of sedentary behavior through messenger-based interventions, so far (Castro et al., 2021; Cotten & Prapavessis, 2016).

Previously, a study evaluating a messenger-based intervention measuring sedentary time subjectively was conducted by the same research group. Promising results of the reduction in sedentary behavior were detected in the intervention group (Kellner & Faas, 2022). Since subjective reporting of sedentary behavior holds certain biases, the study was replicated with an objective measurement method to adequately assess sedentary time.

The primary aim of the current study was to investigate, if messages sent to a smartphone can reduce sedentary time of university students. As a secondary aim, the content and style of the messages was evaluated.

## Methods

The study was designed as a pilot randomized controlled trial with a parallel-group design, where participants were randomly assigned to either intervention (IG) or control group (CG) and objective measurement of sedentary behavior was conducted before (T0) and after the 3-week intervention (T1). The randomization was carried out computer-assisted to assign the participants to either IG or CG. Control group was designed as a waiting list control group. Therefore, subjects of CG received the treatment after the end of the post measurement phase. For the objective measurement of sedentary behavior activPal accelerometers (Pal Technologies Ltd., Glasgow) were used. ActivPAL is a thigh-worn inclinometer with high validity (O’Brien et al., 2022). It registers the inclination of the thigh and can distinguish between sitting/lying, standing, sit-to-stand and stand-to-sit transitions and step counts (Aminian & Hinckson, 2012).

### Participants

After receiving a positive ethics vote by the ethics committee of the faculty of behavioural and cultural studies at Heidelberg University (AZ Kel 2021 1/1), participants were recruited from three different higher education institutions in Heidelberg, Germany (Heidelberg University, Heidelberg University of Education and SRH University Heidelberg). Data protection was carried out according to the General Data Protection Regulation of the European Union.

The participants belong to different fields of study, including Mathematics, Physics, Educational Science, Physical Therapy, Occupational Therapy, Psychology, Special Needs Education, Teacher Training, Health Care and Health and Prevention and therefore show a great variety of students. Nonetheless, the study sample of the pilot study is comparably small, hence no representative conclusions can be stated.

Eligibility criteria included the active participation in one of the three universities, being older than 18 years of age as well as the possession of a smartphone to receive the short messages included in the intervention. Excluded were participants who did not speak sufficient German to understand the messages and those who were physically incapable of standing up. Recruitment was carried out using mailing lists of the three institutions as well as online learning and social media platforms to advertise the study. Interested students enrolled for participation via e-Mail. To secure comparable study conditions for all participants, the data collection and intervention could not be carried out during holidays or the exam phase of the participants. Thus, only a small time period in the middle of the semester was available for data collection of this study. According to the number of available activPal devices, a maximum of 45 students could participate in the study. When subjects collected the measuring device for the pretest, they received verbal and written information about the study, had the opportunity to ask questions, and signed written informed consent. The study was collected in fall 2021, when the covid-situation was still tense. Therefore, most of the participants still attended online classes. As participants were recruited from three different universities, conditions differed between the participants, and it could not generally be assessed whether participants went to university or attended classes online.

### Procedure

After enrollment participants collected the acitvPal device. Participants wore the activPal sensor for 5 days (Monday-Friday) but the first and last day of measurement were omitted because sensors were not worn for 24 h on those days. This left 3 valid days (a valid measurement day must consist of a 24-hour period of wear-time) for data analysis. After the pre-measurement, computer-assisted randomization into IG and CG was performed.

Participants were told to which group they were assigned to since the IG-group needed to sign up for a messenger-app called “notify” to receive the messages. Subjects registered for the news channel in the app with their smartphone after baseline measurement.

Participants in the IG received two messages daily (in the morning and in the evening) which contained prompts to interrupt sitting time and suggestions for isotemporal substitution of sedentary behavior. The CG was designed as a waiting-control group and received the messages for 3 weeks after the post measurement.

To reduce the participants’ sedentary time and increase non-sedentary behavior in the style of light-intensity physical activity (LIPA), the messages were designed based on the theory of action- and coping planning. Strategies integrated into the messages included knowledge transfer, self-observation tasks, goal setting, if-then plans, and environmental restructuring cues. The text messages were linguistically adapted by students for their own peer group in order to address the target group appropriately. The translated messages can be seen online in Supplement 1.

Once the intervention was completed, an evaluation of the intervention-messages was conducted. Therefore, subjects answered six questions concerning the intervention.

### Data Collection and Measures

The primary aim was to measure the sedentary time of the participants before and after the intervention. This was done using activPal devices (Pal Technologies Ltd., Glasgow).

The secondary aim of the study was the evaluation of the messages, which were sent to the intervention group for 3 weeks. The six questions included feasibility of the tasks, consequent adherence to tasks, if the tasks were fun, if the frequency and lengths of the messages was appropriate and if the tasks were executed even after the end of the intervention. Furthermore, they were asked whether they subjectively thought that they had reduced their sitting time. There was also the opportunity for leaving any comments. The answer format was a 5-point Likert scale with the options of fully disagree, somewhat disagree, neither agree nor disagree, somewhat agree, and fully agree.

### Data & Statistical Analysis

The initial convenience sample included 41 students (IG 21, CG 20). In the intervention group, 5 subjects dropped out (T0: 2; T1: 3), in the control group the drop out comprised 2 participants (T0:0; T1:2). Reasons for dropouts were faulty measuring devices, non-participation or dropping out of university during the intervention (see CONSORT flow-diagram 1). Consequently, the final dataset consisted of 34 subjects – 16 in the IG and 18 in the CG. The data was collected on three valid days (a valid day is a full 24-hour period of wear-time) of wearing the activPal device, the average data of those three days is presented here.

Data analysis was carried out using IBM SPSS Statistics 27. Descriptive Data such as Mean ± Standard Deviation (M ± SD) was reviewed to explore differences of the variables from pre- to post- measurement. Unpaired t-test was used to analyze the differences between IG and CG at pretest T0 to check if group randomization was successful. To analyze within- and between differences, single analysis of variance in a mixed design (mixed ANOVA) was carried out in primary outcome variables. Level of significance was set at p < .05 and effect sizes were calculated using a partial eta square (η²).

For further explorative analysis, the change in sedentary time (CST) between T0 and T1 was calculated for each group. Differences in change between both groups were evaluated using unpaired t-test. Moreover, it was investigated if pre-intervention sedentary time was related to the amount of change of sedentary time. A correlation between these values was calculated using the Pearson’s correlation coefficient. For the analysis of the secondary aim, which was the evaluation of the messages, the median (Md) score was calculated for each question.

**Fig. 1:**
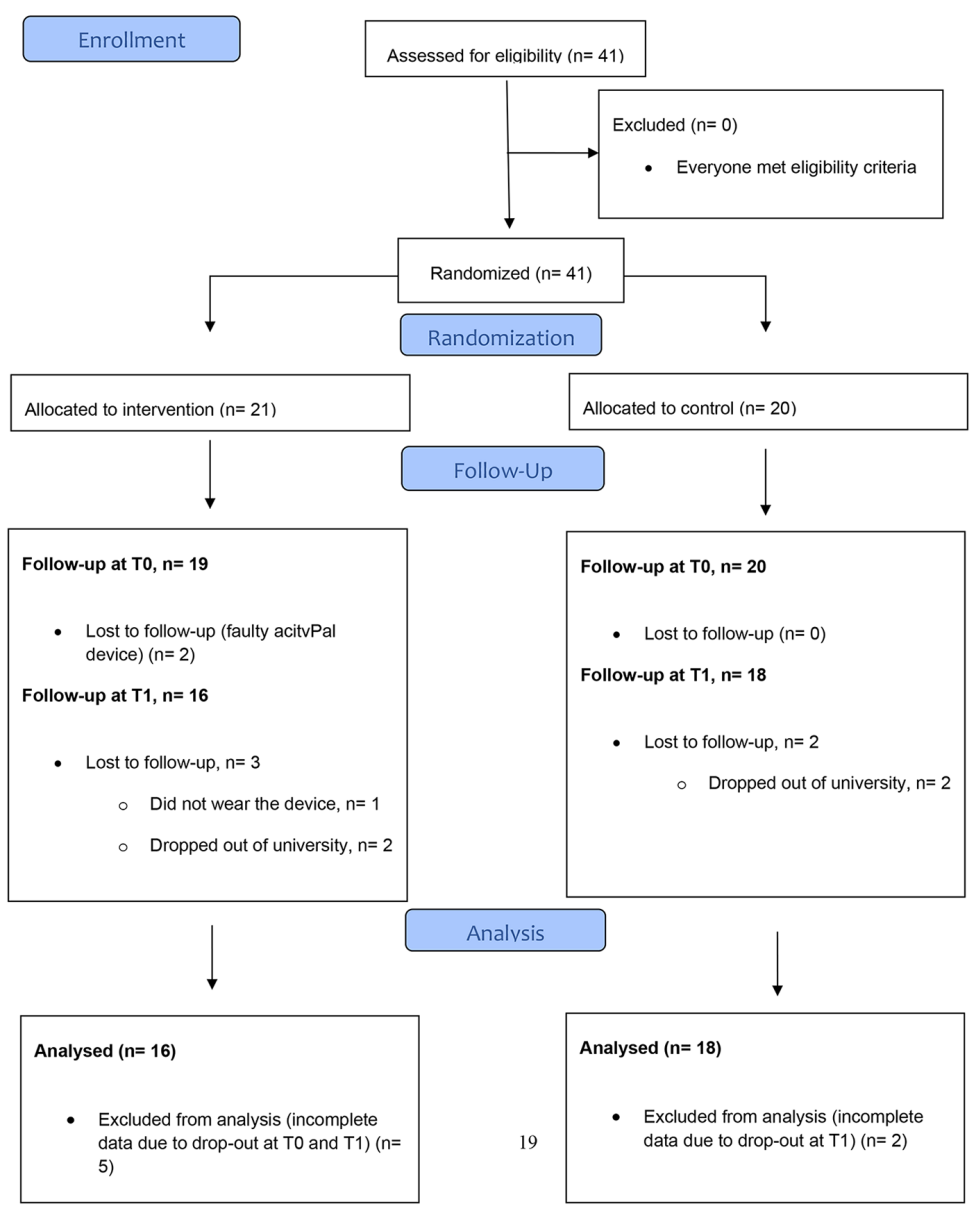
Participant flow-diagram

## Results

### Sample Demographics

The mean age was 22.31 (SD ± 2.59) and 79.4% of the subjects were female. Participants were university students of Mathematics, Physics, Educational Science, Physical Therapy, Occupational Therapy, Psychology, Special Needs Education, Teacher Training, Health Care and Health and Prevention. On average the subjects were in the third Semester of their studies (M = 3.25; SD ± 1.97 range 1–9). Baseline demographics as well as characteristics for each group can be seen in Table 1. Two-sample t-test shows that randomization was successful with no significant baseline differences between the groups IG and CG in sedentary time.

**Table 1:** Baseline characteristics of each group

	Intervention group (N = 16)	Control group (N = 18)
**Sex**		
*** Female***	68.8%	88.9%
*** Male***	18.8%	11.1%
**Age [Mean ± SD]**	22.64 ± 2.27	22.05 ± 2.86
**Semester [Mean ± SD]**	3.64 ± 1.8	2.94 ± 2.07
**Sedentary time** **[min; Mean ± SD]**	586.03 ± 135.4	560.23 ± 111.2

**Table 2:** Descriptive Statistics as well as results from single analysis of variance in a mixed design (interaction effect (time*group))

	Sedentary time [Mean ± SD]		Significance	Effect size
	Pretest	Posttest	p	partial η²
**Overall sample (N = 34)**			.119	.07
***Intervention Group (N = 16)***	586.03 ± 135.4	526.57 ± 79.73		
***Control Group (N = 18)***	560.23 ± 111.19	558.38 ± 126.88		

### Primary Aim

Primary Outcome measurement consisted of the sedentary time of the subjects. Participants in the intervention group showed sedentary time of M = 586.03 (SD ± 135.4) minutes, the control group of M = 560.23 (SD ± 111.2) minutes per day. Throughout the intervention, sedentary time decreased in the intervention group by M = 59.46 (SD ± 102.68) minutes. The mean time change in sedentary behavior in the control group, however, almost stayed the same with a decrease of M = 1.85 (SD ± 106.42) minutes.

A significant time effect in the intervention group can be reported (F (1, 15) = 5.36, p = .03 partial η² = 0.26, whereas a significant interaction effect between the groups could not be detected: F (1, 32) = 2.56, p = .119, partial η² = 0.074.

**Fig. 2:**
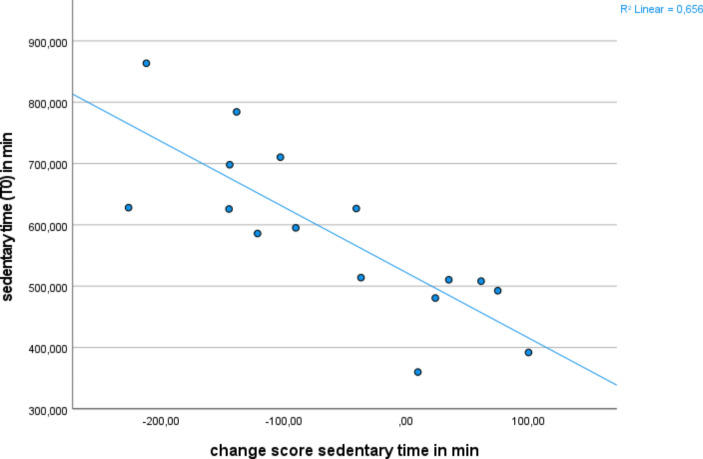
The graph shows the correlation between the measured sedentary time at baseline (Y) and the calculated change of sedentary time during the 3 week-intervention (X) in the intervention group

A large negative correlation between sedentary behavior at baseline and the change of sedentary behavior over time with Pearson’s r = − .81 and p < .001 could be found in the intervention group (Fig. 2). In the control group, no correlation could be detected (Pearson’s r = − .321, p = .19).

### Secondary Aim

Participants of the IG (n = 16) answered the questions about the messages using a 5-point Likert scale. Only participants of the intervention group received the messages, wherefore the sample size for this outcome decreased to n = 16. Most participants agreed that the messages were of good length (Md = 5.0) and frequency (Md = 5.0) and that the tasks were feasible (Md = 4.0) and fun to do (Md = 4.0). Participants admitted that adherence to consequent execution of the tasks was not always given (Md = 2.0). Regarding a long-term implementation, only a minority of the participants fully agreed on maintaining the tasks. Most of the participants stated, that the perpetuation of the tasks without the reminder of the intervention is unlikely (M = 3.0).

The open comments section was used by several participants stating that the study raised their awareness about how long they sit each day.

## Discussion

The aim of the study was to reduce sedentary behavior of university students using a messenger-based intervention. To raise the opportunity for action, the messages were created using the strategies of knowledge transfer, self-observation tasks, goal setting, if-then plans, and environmental restructuring cues. The inclusion of those strategies in the messages should help the participants with action- and coping planning throughout the intervention.

The current study also serves as a pilot study to evaluate the created messages. Overall, it was tested, if this kind of intervention is suitable for health promotion in the target group of university students.

The average reduction of almost one hour of sedentary time in the intervention group is a beneficial effect, while the average sedentary time in the control group hardly changed. Considering the evidence, that all-cause mortality increases by 5% for every additional hour above a daily sitting time of more than seven hours (Chau et al., 2013), the decrease by one hour is of great relevance. Remarkably, a prior study using the same text-messages also shows a decrease in sedentary time of about one hour in the intervention group (Kellner & Faas, 2022). Thus, the prior results, measured with a questionnaire, could now be verified in the current study using objective measurement methods.

The overall sample shows high sedentary times at baseline measurement. With an average of 572.37 (SD ± 121.96) minutes (9.5 h/d) of daily sedentary time the results agree with previously reported data about sedentary behavior in young adults in Germany (Froböse & Wallmann-Sperlich, 2021). These long hours of sedentary behavior are alarming and indicate that university students are at increased risk of detrimental health outcomes. The large standard deviation of around 2 h indicates a large variation between participants. On the one hand, this might be caused by some students only participating in online courses and others in a mixture of online courses and courses in presence. On the other hand, a general heterogeneity of university students must be considered.

Regarding the underlying sample, subjects in the intervention group reduced their sedentary time throughout the intervention from M = 9.7 to 8.7 h per day which brings them near to the cut-off value for hazardous sedentary time (Schmid et al., 2015). However, they are still within the critical range, in which health problems might be the consequence. During post-intervention measurement participants knew which group they had been assigned to, therefore the participants of the intervention group might have subconsciously moved more as they thought this was expected of them. The messenger-based intervention presented only addresses the individual behavior of participants. Environmental conditions, which are of great heterogeneity in the different institutions at university could not be considered. The sedentary behavior evolving from daily university tasks, such as attending classes or writing term papers cannot be mitigated, as they present mandatory tasks at university. Also, in everyday university life, students are bound by the social norm of traditional sitting pedagogy and are exposed to a spatial design mostly designed for sedentary behavior. In order to support students in changing their sitting behavior, a sensitization of lecturers and a movement-friendly room design are desirable in the university setting. Studies in office contexts already indicate that a combination of behavioral and environmental approaches such as implementation of standing desks can be considered promising (Becker et al., 2019; Nguyen et al., 2020). However, online teaching, which was widely available at the time of the study, gave students the opportunity to interrupt periods of sitting, move around during lectures, and set up impromptu standing workstations while studying at home. Eventually, it is not known, how many of the participants followed the advice to do so. However, further exploratory analysis reveals that the participants with the highest sedentary times seem to benefit the most from the intervention, as they reduced sedentary behavior the most. This change was substantially more evident in the IG than in the CG indicating that the intervention was effective.

The evaluation of the messages showed that they were of good length and frequency and that most of the tasks were feasible. Additionally, participants claimed that by receiving the messages each day, they were made aware of their sedentary behavior. Nevertheless, most of the participants admitted, that a consequent execution of the tasks after the end of the intervention is not very likely. Also, as messages were always sent out at the same time in the morning and in the evening of every day, some participants were not quite content with the moment they received the messages. To fulfil the demands of the very heterogeneous target group, a successful tailoring of the intervention, which involves technical and content-related adaptions, must be implemented in the future. It must be noted, that only the participants of the intervention group were able to evaluate the messages, as participants in the control group did not receive any messages. Thus, the sample size for the secondary aim – the evaluation of the feasibility of the intervention – was reduced to n = 16.

However, to counteract long periods of sitting in the context of face-to-face teaching, a multi-component concept as described in the Heidelberg Model of Physically Active Teaching can be a promising approach. The components of the model include activity permissible room design, physically activating teaching methods, movement breaks, curricular-based study offers in physical activity promotion and lecturer training as it focuses on teachers as facilitators of movement (Rupp et al., 2022).

### Strengths and Limitations

As prior investigations show the highest validity for thigh-worn objective accelerometers and a substantial underestimation of subjectively measured sedentary time (Aunger & Wagnild, 2022; Urda et al., 2017), the underlying study used activPal devices to adequately assess the sedentary behavior of the population. The use of objective measurement methods mitigates errors such as recall bias or bias of social desirability. However, there is still a chance that wearing the device in addition to the knowledge about the assigned group during T1 measurement reminded the participants that their behavior was controlled, which might have made them move more than usual (Hawthorne-effect). Also, the small sample with predominantly female participants was not representative for all students at the universities, which must be considered as a limitation of the investigation. Regarding the evaluation of the messages, the results represent the opinion of 16 students which might not be generalized to all students. Nevertheless, the objective measurement has very high validity and reliability, which presents a great advantage of the underlying investigation.

## Conclusion

In conclusion, sedentary times are on an extraordinary high level for the majority of the sample, which induces a call to action for more interventions addressing sedentary behavior of university students. The current pilot study appears to have an impact on the population with the highest sedentary times, but this needs further investigation. Nonetheless, those findings should be considered, when further research is being conducted. All in all, more research regarding mobile-based interventions for the reduction of sedentary behavior should be conducted.

## Data Availability

Not applicable.
